# Effects of alcohol on the symptoms of gouty arthritis and taxonomic structure of gut microbiota in C57BL/6 mice

**DOI:** 10.3389/fmicb.2023.1257701

**Published:** 2023-09-13

**Authors:** Yu Feng, Haihui Sun, Ruilou Zhu, Jianxing Tao, Rui Su, Yundong Sun, Dawei Wang

**Affiliations:** ^1^Department of Orthopedic, Shandong Provincial Hospital Affiliated to Shandong First Medical University, Jinan, China; ^2^Department of Cardiology, Shandong Provincial Hospital Affiliated to Shandong First Medical University, Jinan, China; ^3^Department of Clinical Laboratory, Shandong Provincial Hospital Affiliated to Shandong First Medical University, Jinan, China; ^4^Key Laboratory for Experimental Teratology of Ministry of Education, Department of Microbiology, School of Basic Medicine, Cheeloo College of Medicine, Shandong University, Jinan, China

**Keywords:** alcohol, gouty arthritis, gut microbiota, purine metabolism, pro-inflammatory cytokine

## Abstract

Gout is an acute arthritis caused by the elevated levels of serum uric acid (UA), and its prevalence has been rapidly increasing. Alcohol abuse could lead to a series of health problems. Multiple pieces of evidence suggest that alcohol intake affects the development and progression of gout, while the gut microbiota plays an important role in the development of gout and the long-term alcohol consumption could affect the stability of the gut microbiota. This study aimed to explore the effects of alcohol intake at different concentrations on gouty arthritis based on the gut microbiota. We investigated the effects of different concentrations of alcohol on gouty arthritis in mouse models of acute gouty arthritis established by injection of monosodium urate (MSU) crystals into C57BL/6 mice. The results indicated that the high-alcohol consumption not only exacerbated joint swelling and pain, increased the levels of UA, tumor necrosis factor-α (TNF-α), interleukin-1β (IL-1β), and interleukin-6 (IL-6), but also showed dramatic effects on the composition and structure of the gut microbiota in gouty mice. Two key microorganisms, *Parasutterella* and *Alistipes*, could aggravate gout symptoms through lipopolysaccharide biosynthesis, riboflavin metabolism, phenylalanine metabolism, and arginine and proline metabolisms. In conclusion, our study suggested that high-concentrations of alcohol altered the gut microbiota structure in gouty mice induced by MSU crystals, which could exacerbate gouty symptoms by enhancing pro-inflammatory pathways.

## 1. Introduction

Gout is an acute arthritis caused by elevated serum uric acid (UA) levels, activation of inflammatory mediators, and cytokine release, ultimately leading to deposition of monosodium urate (MSU) crystals in the joints (So and Martinon, 2017). In addition, gout can lead to kidney diseases such as uric acid nephrolithiasis and chronic nephropathy (Singh and Gaffo, 2020). In recent years, the prevalence of gout has been rapidly increasing worldwide due to the changes in dietary habits and lifestyle (Kuo et al., 2015). The overall estimated prevalence of gout is 2.6%, with a significantly higher proportion in males (3:1 to 4:1) (Ankli and Daikeler, 2023). Alcohol is generally considered a potential risk factor for acute gouty arthritis and recurrent gout attacks (Neogi et al., 2014).

Alcohol abuse could lead to a range of health problems (Barbería-Latasa et al., 2022). For example, it has been reported that an increase in alcohol consumption is closely associated with an increased risk of gout, with a 1.17-fold increase in risk for every 10 grams of alcohol consumed per day (Tu et al., 2017). A cross-sectional study has shown that gout patients with consumption of 15–30 g and 30–60 g of alcohol per day gained increased risks of recurrence by 1.36-fold and 1.51-fold, respectively, compared to non-drinking gout patients (Neogi et al., 2014). Consumption of various alcoholic beverages, e.g., liquor, beer, and wine, could increase the UA levels and thereby increase the risk of gout attacks (Neogi et al., 2014). Furthermore, some of the non-alcoholic components in alcoholic beverages may contain different purine contents, thus excessive alcohol consumption could induce the progression of gouty arthritis by providing excessive exogenous purines (Liu et al., 2021). However, the molecular mechanisms underlying the exacerbation of gouty arthritis by alcohol are still not clearly known.

Studies have found that the homeostasis of the gut microbiota influences the development of human physical and mental health (Postler and Ghosh, 2017), while the gut microbiota could influence the progression of gouty arthritis (Guo et al., 2016). Recent research has shown that *β*-carotene and green tea powder diet could alleviate gout symptoms by modulating the structure of the gut microbiota in gouty mice (Feng et al., 2022). Furthermore, studies have shown that alcohol could increase the diversity of gut microbiota in mice and cause damages in both hepatic and colon tissues (Wang et al., 2018). Moreover, strong evidence has suggested that gut microbiota composition in alcohol abusers greatly differs from that in healthy individuals (Dubinkina et al., 2017).

Dietary habits are important factors affecting the composition and function of gut microbiota (Qin et al., 2022). Alcohol abuse could alter gut permeability, leading to bacterial translocation to mesenteric lymph nodes and hepatic tissue, further exacerbating the disease progression (Leclercq et al., 2014; Wang et al., 2016). Therefore, we hypothesized that alcohol could alter the structure of the gut microbiota in gouty mice, which could exacerbate the progression of gout. In this study, our goals were to establish a mouse model of acute gouty arthritis by injection of MSU crystals and to further investigate the effects of alcohol at different concentrations on gout symptoms, pro-inflammatory cytokines, and gut microbiota in mice. The results showed that high concentrations of alcohol changed the structures of the gut microbiota and aggravated gout symptoms in gouty mice, whereas the significant effects of low concentrations of alcohol on mice with gout were not observed.

## 2. Materials and methods

### 2.1. Consumable alcohol and MSU crystals

Consumable alcohol (ethanol and water, 98–99% of total) was purchased from Beijing Hongxing Co. (Beijing, China). MSU was purchased from Sigma Aldrich Trading Co. (Shanghai, China; Cat. U2875).

A total of 800 mg of MSU was accurately weighed and added into the boiling Milli-Q water (155 mL) with the pH of the solution adjusted to 7.2 using both NaOH (5 mL) and hydrochloric acid, and gradually cooled with stirring at room temperature. Then, MSU crystals were colledted by centrifugation at 3000 g and 4°C for 2 min (Model 5430R, Eppendorf, Germany) and stored after sterilization at 180°C for 2 h (Ruiz-Miyazawa et al., 2017).

### 2.2. Animals

Thirty Specific Pathogen Free (SPF) C57BL/6 male mice (4 weeks old with average weight = 15 ± 2 g) were purchased from Beijing Vital River Experimental Animal Technology Co., Ltd. (Beijing, China). All mice were continuously provided with food and water in a standard environment (12/12 h light/dark cycle, 40% humidity, and temperature of 22 ± 1°C). During the entire experiment (6 weeks), there were no deaths or other abnormal conditions observed in any of the mice. The Animal Ethics Review Board of the Provincial Hospital of Shandong First Medical University approved this study (Permit No. 2022–025).

### 2.3. Experimental design

**Figure 1:**
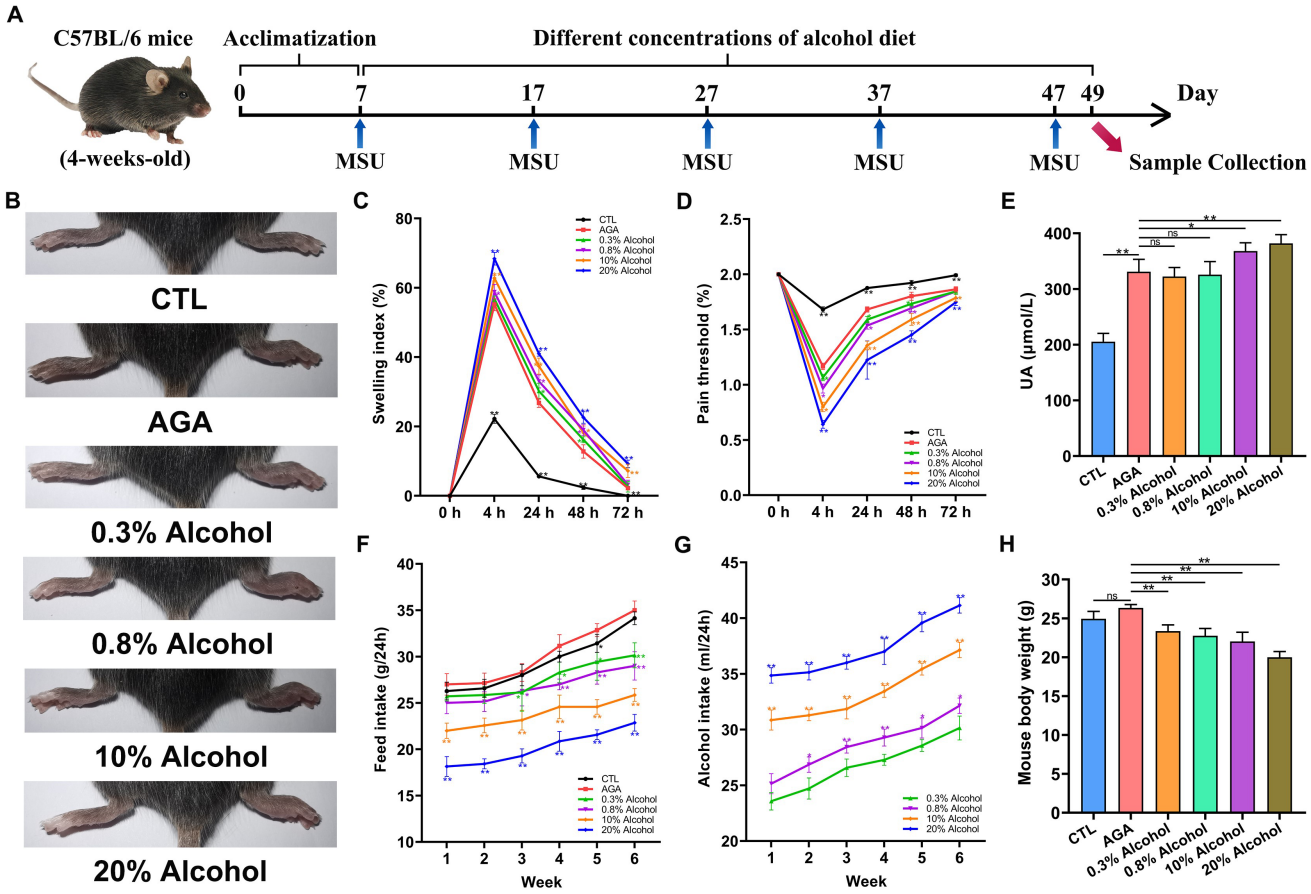
Effect of alcohol on body weight and foot joint gout symptoms in mice. **(A)** Schematic diagram of the experimental treatments. MSU, monosodium urate. **(B)** Schematic representation of the appearance of foot joints in six groups of mice. CTL, control. AGA, acute gouty arthritis. **(C)** Comparison of changes in footpad swelling in six groups of mice. **(D)** Comparison of changes in footpad pain thresholds in six groups of mice. **(E)** Comparison of changes in serum uric acid (UA) levels in six groups of mice. **(F)** Food intake of the six groups of mice. **(G)** Comparison of changes in alcohol intake in mice of the alcohol group. **(H)** Comparison of body weight changes in six groups of mice after 6 weeks of feeding. Data are presented as mean ± standard deviation. Statistical differences are determined by *p* < 0.05 (*) and *p* < 0.01 (**), respectively; “ns” represents no statistical significance.

After 1 week of rearing in a standardized environment, 30 mice were randomly and evenly grouped and kept in 6 cages (i.e., 5 mice per cage), including the healthy control group (CTL), the acute gouty arthritis model group (AGA), the two low-concentration alcohol experimental groups (treated with 0.3 and 0.8% alcohol, respectively), and the two high-concentration alcohol experinmental groups (treated with 10 and 20% alcohol, respectively). Previous clinical studies have confirmed a correlation between daily alcohol consumption in adults and an increased risk of gout (Tu et al., 2017). Therefore, the alcohol concentration consumed by mice in the low concentration alcohol group was calculated based on the ratio of daily alcohol consumption in adults to body weight. The concentration of alcohol consumed by mice in the high-concentration alcohol groups was determined based on previous studies (Wang et al., 2018). All six groups of mice were given a normal diet every day (protein content 18–22% Kcal, fat content 4–8% Kcal, total 3.56 Kcal/g; Wuhan Shisheng Biotechnology Co., Wuhan, China). In the CTL and AGA groups, mice were orally administered drinking water daily, while the mice in the alcohol groups were given alcohol orally. Additionally, every 10 d, mice in the CTL group were injected with 40 μL of PBS into the right hind paw pad, while 1 mg of MSU crystals mixed with 40 μL of PBS was injected into the right hind paw pad of the other five groups of mice (Figure 1A; Lin et al., 2020). During the entire experiment (6 weeks), both food and alcohol bottles were weighed every morning at 10 am to calculate the food and alcohol intakes by the mice.

### 2.4. Evaluation of foot joint hypersensitivity and oedema

Monosodium urate crystals were injected into the right hindfoot pads of mice at 10 a.m. each day. Starting from the second injection of MSU crystals, the thickness of the right hindfoot pads of all mice was measured using digital calipers (Meinaite, Germany) before and at 4, 24, 48, and 72 h after injection. The mechanical withdrawal threshold (MWT) was quantified using von Frey monofilaments (Danmic Global LLC Co., Ltd., USA) to assess the foot pain. Foot swelling was expressed as the ratio of Δmm/mm (baseline) of the joint (Lin et al., 2020).

### 2.5. Sample collection

At the end of the 6 week (42 day) experiment, all mice were weighed and samples were collected. The mouse blood was collected through the orbital vein, then centrifuged to collect serum and stored at −80°C. Subsequently, all mice were exectued using CO_2_ (100%) for 5 min, and fresh fecal samples were immediately collected from mouse colon tissues that were stored at −80°C. In addition, the hepatic and right foot joint tissues in mice were collected in an ice water bath environment and stored at −80°C after rapid freezing in liquid nitrogen.

### 2.6. Measurements of cytokines and enzymes

The mouse foot joint specimens and hepatic specimens were kept at room temperature for slow thawing, followed by homogenization of the tissues (0.05 g of tissue per 1.0 mL of buffer solution) and centrifugation of the tissues at 6,500 g and 4°C for 10 min to collect the supernatant. Previous studies have shown that xanthine oxidase (XOD) and adenosine deaminase (ADA) in the hepatic could further influence the uric acid production by participating in the purine metabolism (Lou et al., 2020). Therefore, the enzymatic activities of myeloperoxidase (MPO), XOD, and ADA were assayed using a mouse ELISA kit (Kelvin, Suzhou, China). Previous studies have shown that the pro-inflammatory cytokines IL-6, IL-1β, and TNF-α were associated with inflammatory activity in gout patients (Galozzi et al., 2021). The levels of serum UA and proinflammatory cytokines (i.e., IL-1β, IL-6, and TNF-α) in mice as well as in foot joint supernatants were measured by mouse ELISA kits (Novus, Germany).

### 2.7. Histopathological assessment of foot joint

Foot joint tissues of mice were thawed at room temperature and washed with PBS solution. Subsequently, the foot joints were immersed in 10% paraformaldehyde for 12 h and then decalcified with EDTA for 14 d. The decalcified foot joint tissues were embedded in paraffin for sectioning of 5 μm thickness. The sections were then stained with hematoxylin and eosin (HE) and observed for morphological changes and degree of inflammation in the foot joint tissues.

### 2.8. Gut microbiota analysis

Nucleic acids were extracted from mouse fecal samples using the TGuide S96 Magnetic Soil/Fecal DNA Kit (Tiangen Biotechnology Co., Ltd., Beijing, China) according to the manufacturer’s instructions. The 16S rRNA gene was amplified and sequenced on the Illumina Miseq platform using the universal bacterial primer 338F (5’-ACTCCT ACGGGAGGCAGCA-3′) and 806R (5’-GGACTACHVGGGT WTCTAAT-3′) by BioMarker Technologies Co. (Beijing, China) (Zhang et al., 2021). Sequencing libraries were quality checked for high-quality tag sequences. The 16S rRNA sequences were analyzed using QIIME version 1.9.3. Sequences were clustered at 97% similarity level (USEARCH, version 10.0), and OTUs were filtered using 0.005% of the number of all sequences as a threshold. Species annotation and classification were performed based on the Silva database^1^ and RDP Classifier.^2^ Alpha diversity indices were calculated using Mothur (version v. 1.30). Beta diversity analysis was performed by principal coordinate analysis (PCoA) to compare differences in community composition and structure among samples. Finally, biomarker taxa that were statistically different between groups were screened at the genus level. Spearman’s correlation coefficients between microbial communities and gout symptoms were calculated using the “cort.test” function in the R statistical software, and the correlations were visualized in heat maps. In addition, the metabolic functions of the gut microbiota were predicted using Picrust (Feng et al., 2022). These predictions were precalculated based on the gene functional annotation using the Kyoto Encyclopedia of Genes and Genomes (KEGG) database.^3^ The datasets used in this study are deposited in the NCBI database^4^ with the accession number PRJNA990312.

### 2.9. Statistics

Biochemical data were plotted using GraphPad Prism (V8.0.2). Significant differences in microbial structure and gene expressions between two groups were determined using *t*-test based on *p* < 0.05.

## 3. Results

### 3.1. Gout symptoms aggravated in mice with intake of high concentration of alcohol

The effects of different concentrations of alcohol on weights and foot joint gout symptoms in mice were evaluated by measuring the daily food and alcohol intakes, foot pad swelling and pain threshold, levels of serum UA (Figure 1). Compared to the CTL group, the level of foot joint swelling in other groups of mice was significantly higher, the foot pad mechanical pain thresholds were significantly decreased, and the serum UA level were significantly increased (Figures 1B–E). The above indexes indicated that the MSU crystals were injected into the right hind paw of mice to successfully establish an acute gouty arthritis mouse model. In addition, compared to the AGA group, the mice of the alcohol groups were observed with significantly increased degree of foot joint swelling, as the concentrations of alcohol were increased, while the foot pad mechanical pain thresholds were significantly decreased (Figures 1C,D). Compared to the AGA group, the serum UA levels were significantly increased in mice fed with high concentrations of alcohol (Figure 1E).

The results of daily weighing of food and alcohol bottles showed that as the concentration of alcohol was increased, the food intake of the mice was gradually decreased and the alcohol intake was gradually increased (Figures 1F,G). After 6 weeks of feeding, all mice in the alcohol group showed a significant decrease in body weight compared to the AGA group (Figure 1H).

### 3.2. Gout inflammation index aggravated in mice with intake of high concentration of alcohol

**Figure 2:**
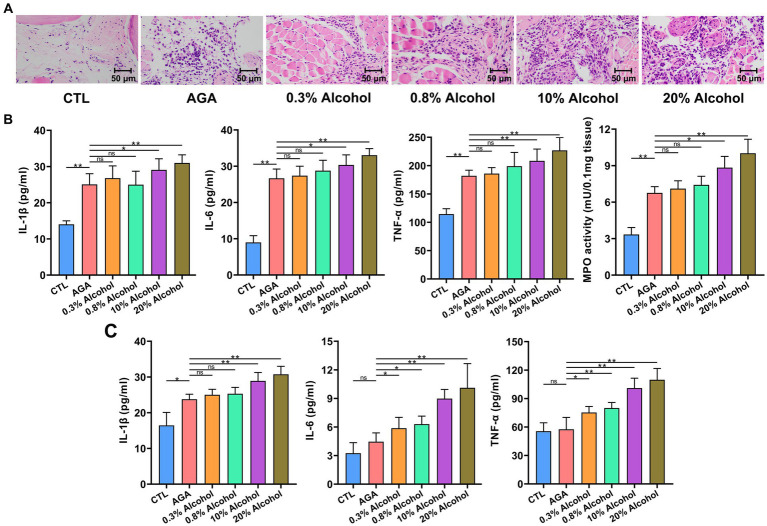
Gout-associated inflammatory markers aggravated by alcohol in mice. **(A)** HE analysis of the right hindfoot pads of six groups of mice. **(B)** Comparison of changes in the levels of cytokines interleukin-1β (IL-1β), IL-6, and tumor necrosis factor-α (TNF-α), and the enzymatic activity of myeloperoxidase (MPO), in foot joint tissues of six groups of mice. **(C)** Comparison of changes in serum cytokine (i.e., IL-1β, IL-6, and TNF-α) levels in six groups of mice (based on ELISA). Data are presented as mean ± standard deviation. Statistical differences are determined by *p* < 0.05 (*) and *p* < 0.01 (**), respectively; “ns” represents no statistical significance.

To further investigate the effects of alcohol on gouty arthritis, the inflammation of gouty sites in mice was evaluated in each group of mice (Figure 2). Histological analysis showed that compared to the CTL group, the AGA group of mice exhibited significantly increased infiltration of inflammatory cells in the foot joints (Figure 2A). Compared to the AGA group, treatment of high concentrations of alcohol dramatically increased the infiltration of inflammatory cells in the foot joints of gouty mice, while the significant changes were not observed in the number of foot joint inflammatory cells of mice treated with low concentrations of alcohol (Figure 2A). Similarly, compared to the AGA group, the high alcohol concentration group showed significantly higher levels of MPO, IL-1β, IL-6, and TNF-α in the right foot joints of mice, and there were no significant changes in the levels of these inflammatory factors in the right foot joints of mice in the low-concentration alcohol group (Figure 2B).

In order to further investigate the changes in inflammatory factors associated with gout, the expression levels of IL-1β, IL-6, and TNF-α were re-examined in serum (Figure 2C). Serum IL-1β levels were significantly higher in gouty mice in the high-alcohol group compared with the AGA group, while no significant changes were detected in mice treated with low concentrations of alcohol. Conversely, as the concentrations of alcohol were increased, the serum of mice in all alcohol groups showed progressively higher levels of TNF-α and IL-6.

### 3.3. Intestinal microbial structure in mice altered by the intake of high concentrations of alcohol

**Figure 3:**
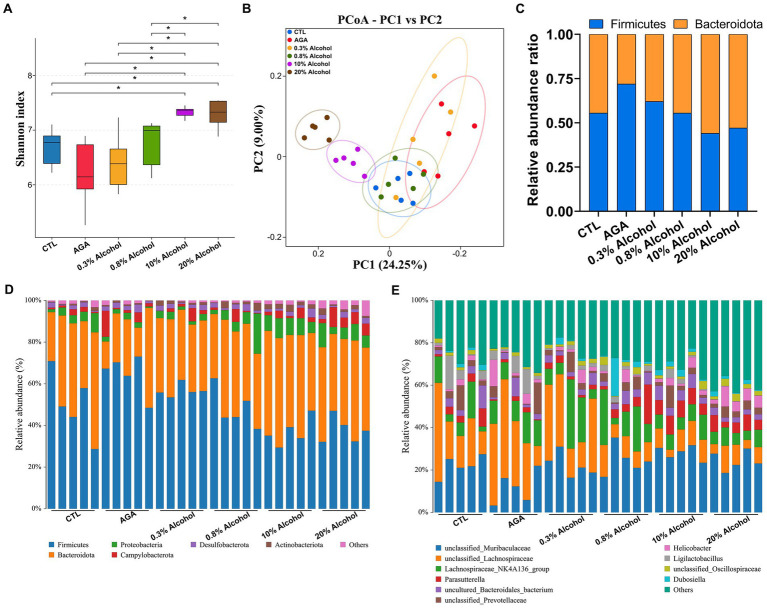
Comparison of changes in gut microbiota in six groups of mice. **(A)** Alpha diversity analysis of the intestinal microbiota in six groups of mice. **(B)** Principal coordinate analysis (PCoA) of intestinal microbiota in six groups of mice. **(C)** Variations in the relative proportions of Firmicutes and Bacteroidota in the intestines of six groups of mice. **(D)** Comparison of changes in the composition of the intestinal microflora at the phylum level in six groups of mice. **(E)** Comparison of changes in the composition of the gut microbiota at the genus level in six groups of mice. Data are presented as mean ± standard deviation. The statistical difference is determined by *p* < 0.05 (*).

The 16S rRNA sequencing was used to analyze fecal samples from gouty mice to study the effect of alcohol on the gut microbiota of mice. The results showed that the microbial alpha diversity of mice was gradually increased as the concentration of alcohol was increased, compared to the AGA group (Figure 3A). The microbial alpha diversity was significantly increased in mice with gout treated with high concentrations of alcohol (Figure 3A; Supplementary Figure S1). PCoA results showed that the gut microbiota of mice in the high alcohol concentration group was significantly different from that of mice in the CTL and AGA groups, while the gut microbiota of mice in the low alcohol concentration group was not significantly different from that of mice in the CTL and AGA groups (Figure 3B). Analysis of the changes in the dominant bacterial groups at the phylum level showed a gradual decrease in the ratio of Firmicutes to Bacteroidota (F/B) as the concentration of alcohol was increased (Figures 3C,D). Similarly, at the genus level, there were significant differences in the structure of the gut microbiota in different groups of mice (Figure 3E). Compared to the AGA group, alcohol consumption in mice resulted in decreased relative abundances of *unclassified_Lachnospiraceae* and *Ligilactobacillusp.,* while the relative abundances of *unclassified_Muribaculaceae* and *Parasutterella* were increased (Figure 3E).

**Figure 4:**
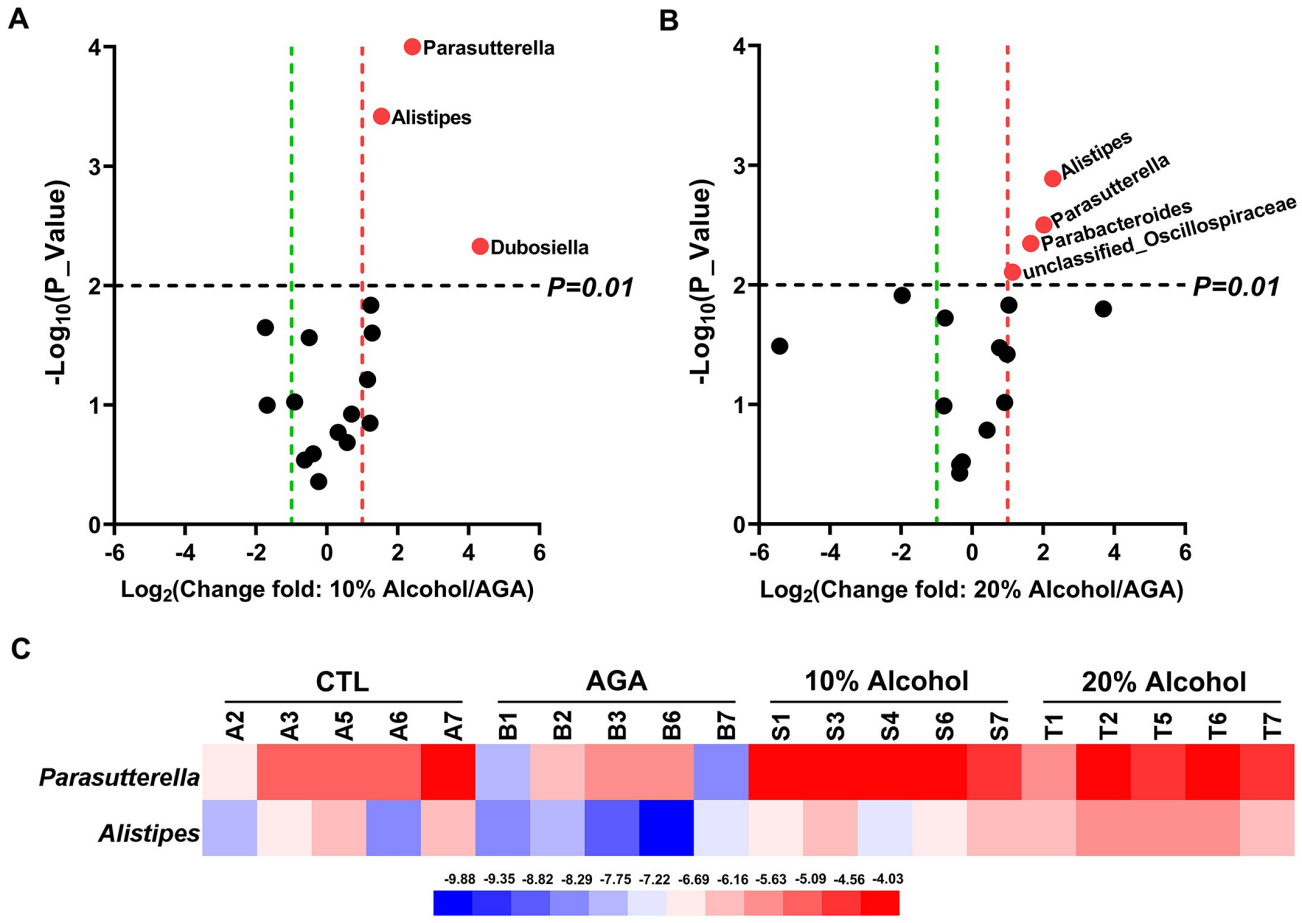
Comparative analysis of changes in the genus level of key bacteria in mice in high alcohol concentration groups. **(A)** Volcano plot analysis of changes in the intestinal microbiota of mice in the 10% Alcohol group compared to the AGA group. **(B)** Volcano plot analysis of changes in the intestinal microbiota of mice in the 20% Alcohol group compared to the AGA group. The significant difference is determined by *t*-test. Bacteria with relative abundance >1% are identified as key bacteria. **(C)** Heatmap showing the relative abundance of the key bacteria co-occurring in both **(A,B)**.

The effects of low concentrations of alcohol intake on the gut microbiota of gouty mice were found to be insignificant by *α*-diversity and *β*-diversity analyses (Figures 3A,B), only the effect of high concentration of alcohol on intestinal microbiota of mice was further explored (Figure 4). At the genus level, the relative abundances of bacteria greater than 1% were compared between the mice of AGA group and the high alcohol concentration group (Figures 4A,B). Compared to the AGA group, the relative abundances of *Parasutterella*, *Alistipes*, and *Dubosiella* were significantly decreased in the 10% alcohol group (Figure 4A), while the relative abundances of *Parasutterella*, *Alistipes*, *Parabacteroides*, and *unclassified_Oscillospiraceae* were significantly increased in the 20% alcohol group (Figure 4B). Therefore, two genera, i.e., *Parasutterella* and *Alistipes*, common to both the 10 and 20% alcohol groups of mice, were identified as the key bacteria. In addition, changes in the relative abundances of critical bacteria in each of the samples from the 4 groups of mice were visualized with the heatmaps (Figure 4C).

### 3.4. Intestinal microbial interactions in mice altered by the intake of high concentration of alcohol

**Figure 5:**
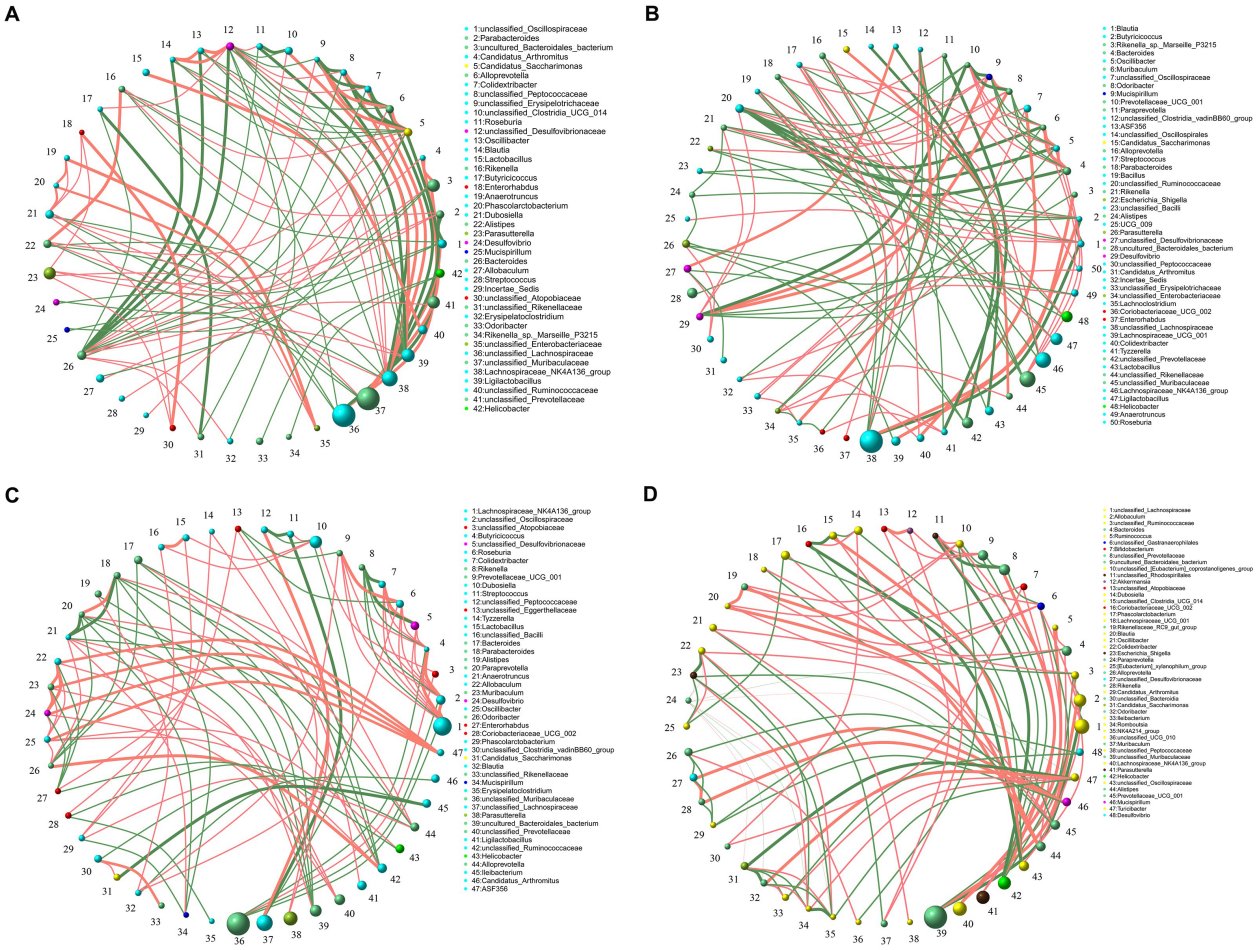
Correlation of microbiota among the top 50 taxa at the genus level in the intestines of four groups of mice, including the CTL group **(A)**, the Model group **(B)**, the 10% Alcohol group **(C)**, and the 20% Alcohol group **(D)**. The size of the circle represents the relative abundance, the line represents the correlation between the two taxa at both ends of the line, the thickness of the line represents the level of the correlation, the orange line represents positive correlation, and the green line represents negative correlation.

Spearman’s correlation ≥0.1 and *p* ≤ 0.05 were used as screening criteria to select the 50 most relatively abundant microbials for further study of their correlation in the gut microbiome.The results showed that the intestinal microbes were closely associated with each other (Figure 5). The interactions among intestinal microbes were increased in mice of AGA group compared to the CTL group (Figures 5A,B). In contrast, administration of high concentrations of alcohol to mice significantly reduced the gut microbial interactions (Figures 5C,D). For example, in the CTL group, *Desulfovibrio* (#24) was negatively correlated with *unclassified_Lachnospiraceae* (#36) and *unclassified_Oscillospiraceae* (#1) (Figure 5A). *Desulfovibrio* (#29) was negatively correlated with *Bacteroides* (#4), *Oscillibacter* (#5), *Muribaculum* (#6), and *Prevotellaceae_UCG_001* (#10) in the AGA group (Figure 5B). In the 10% alcohol group, *Desulfovibrio* (#24) was negatively correlated with *Enterorhabdus* (#27) (Figure 5C). In the 20% alcohol group, *Desulfovibrio* (#48) was negatively correlated with *Candidatus_Arthromitus* (#29) and *Ileibacterium* (#33) (Figure 5D). The most pronounced decrease in interactions between gut microbes was observed in mice treated with 20% alcohol (Figure 5D).

### 3.5. Metabolic functions of the intestinal microbiota in mice altered by the intake of high concentration of alcohol

**Figure 6:**
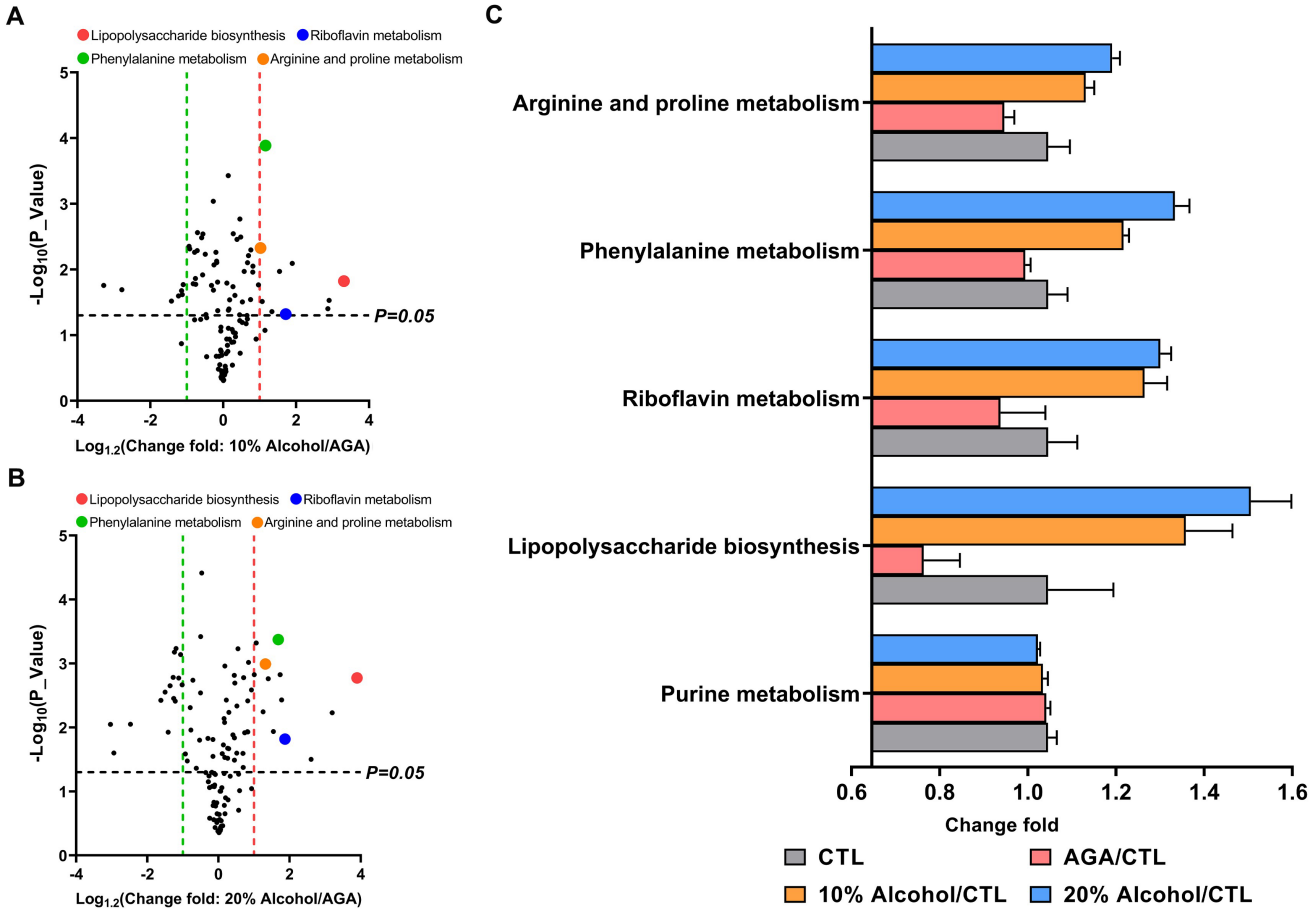
Comparison of the relative abundances of functional profiles of the intestinal microbiota of mice in high alcohol concentration groups based on Picrust. **(A)** Volcano plot analysis of altered KEGG pathways in mice of the 10% Alcohol group vs. Model group, with mean relative abundance >0.1%. **(B)** Volcano plot analysis of altered KEGG pathways in mice of the 20% Alcohol group vs. Model group, with mean relative abundance >0.1%. The significant difference is determined by *t*-test. **(C)** Relative abundance of five key metabolic pathways identified in both **(A)** and **(B)** showing the significant difference in the four groups of mice. Data are presented as mean ± standard deviation.

Gut microbiota functions in mice were predicted by PICRUSt based on the KEGG database (Figure 6). The results indicated that the levels of the four metabolic pathways, compared to the AGA group, namely, lipopolysaccharide biosynthesis, riboflavin metabolism, phenylalanine metabolism, and arginine and proline metabolism, were significantly elevated in the gut microbiota of mice in the high concentration alcohol group (Figures 6A,B). The levels of purine metabolic pathways, which are closely related to uric acid levels, were not significantly increased (Figure 6C). The results of activities of two enzymes (i.e., XOD and ADA) related to purine metabolism in the hepatic tissue revealed no significant changes in the hepatic enzyme activities of mouse XOD and ADA in the high-alcohol group compared to the AGA group (Supplementary Figure S2).

### 3.6. Correlations among key gut microorganisms, inflammatory indicators, and metabolic pathways in mice

**Figure 7:**
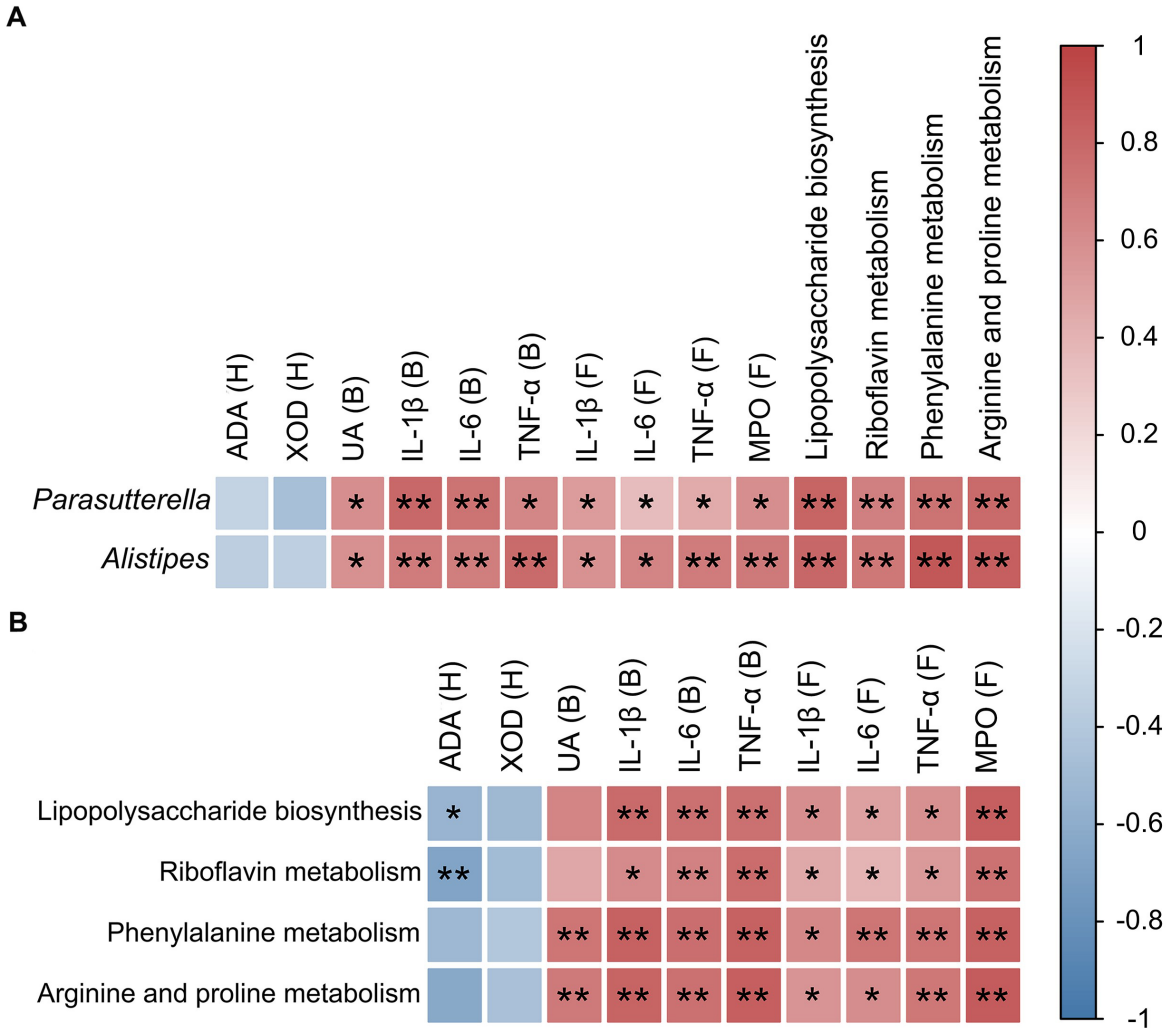
Correlation analyses between microorganisms, pro-inflammatory cytokines, enzymes, and metabolic pathways in mice. **(A)** Correlation between key intestinal microorganisms (i.e., *Parasutterella* and *Alistipes*) and pro-inflammatory cytokines, enzymes, and metabolic pathways. **(B)** Correlation between metabolic pathways and both pro-inflammatory cytokines and enzymes. The relative R values range from −1 to 1 (i.e., blue to red). B, blood; H, hepatic tissue; F, foot joint tissue. Statistical differences are determined by *p* < 0.05 (*) and *p* < 0.01 (**), respectively.

Spearman rank correlation coefficient heatmap analysis was applied to analyze the association between key bacterial taxa, pro-inflammatory factors, serum UA, foot and joint MPO, hepatic XOD, and ADA levels, and four key metabolic pathways (Figure 7). The results showed that the two key bacterial genera, *Parasutterella* and *Alistipes*, were not correlated with XOD and ADA activity levels, but were significantly and positively correlated with the levels of IL-1β, IL-6, TNF-α, UA, and MPO (Figure 7A). Both *Parasutterella* and *Alistipes* were also positively correlated with four metabolic pathways, i.e., lipopolysaccharide biosynthesis, riboflavin metabolism, phenylalanine metabolism, and arginine and proline metabolism (Figure 7A), while both lipopolysaccharide biosynthesis and riboflavin metabolism were positively correlated with the levels of IL-1β, IL-6, TNF-α, and MPO, negatively correlated with the level of ADA, and not correlated with XOD and UA levels (Figure 7B). Phenylalanine metabolism as well as arginine and proline metabolism were not significantly correlated with XOD and ADA levels, but were positively correlated with UA, IL-1β, IL-6, TNF-α, and MPO levels (Figure 7B).

## 4. Discussion

**Figure 8:**
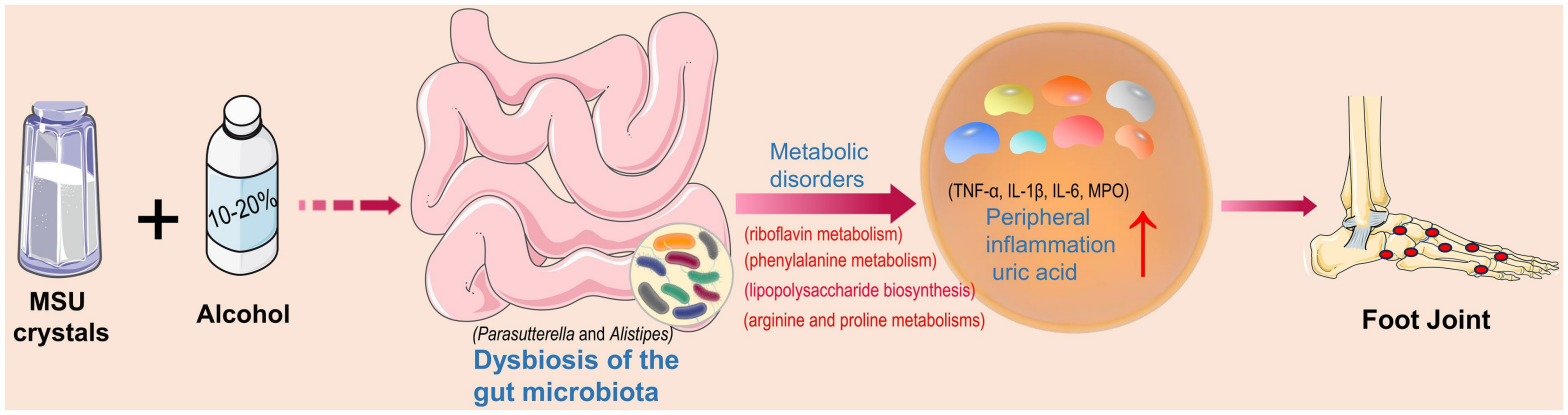
Intestinal bacterial disorders and abnormal metabolic pathways as well as increased levels of pro-inflammatory factors and uric acid caused by alcohol and monosodium urate (MSU) crystals to exacerbate gout symptoms.

Acute gout attacks could severely affect people’s physical and mental health (Terkeltaub, 2017). Strong evidence has suggested a close relationship between alcohol consumption and the development of gout, with intestinal microbiota playing an important role in gout (Neogi et al., 2014; Guo et al., 2016). The results of this study showed that treatment of high concentrations of alcohol exacerbated the acute gout symptoms induced by MSU crystals in mice and altered the gut microbial composition of gout mice. It is commonly known that changes in gut microbiota could lead to metabolic disorders. Our results showed that the production of abnormal metabolites enhanced the gout symptoms by promoting the release of inflammatory mediators (Figure 8).

Alcohol is an addictive substance consumed worldwide, and the alcohol consumption levels and patterns are closely associated with various types of chronic diseases (Hendriks, 2020). Many studies have suggested that moderate drinking could lower the risk of death, mainly due to reduced risks of cardiovascular diseases and diabetes (Larsson et al., 2016; Li et al., 2016). However, chronic alcohol abuse could cause alcohol use disorders and increased risks of various types of cancers and neurological diseases (Larsson et al., 2013; Hendriks, 2020). Currently, there are still a large number of significant controversies surrounding the safe intake levels of alcohol (Barbería-Latasa et al., 2022).

### 4.1. High concentration alcohol intake alters the structure of gut microbiota in mice

Gut microbiota, generally considered an endocrine organ, plays an important role in regulating the host immune system and maintaining the intestinal barrier integrity (Kogut et al., 2020). A healthy gut microbiota promotes host growth and development, whereas alterations in the structure of the gut microbiota could have many detrimental effects on the host (Compare et al., 2016). Furthermore, research has shown that the long-term alcohol consumption could affect the stability of the gut microbiota (Dubinkina et al., 2017), while alcohol-impaired mice have been revealed with a significantly increased diversity of gut microbiota, with varied degrees of damage to both the hepatic and colon tissues (Wang et al., 2018). Moreover, alcohol could induce osteoporosis in mice by regulating variations in the gut microbiota to further activate osteoclasts (Cheng et al., 2021).

Studies have shown that MSU crystals could alter the structure of the gut microbiota (Vieira et al., 2015). Furthermore, previous studies revealed significant changes in gut immunity and environment during the acute and recurrent phases of arthritis (Nemoto et al., 2020). Our results showed that as the concentrations of alcohol consumed by gout mice were gradually increased, the alpha diversity of gut microbiota in gout mice was also gradually increased, while the relative abundance of Firmicutes was decreased. Firmicutes is generally considered an anti-inflammatory bacterium (Natividad et al., 2015). The imbalance of the F/B ratio may lead to metabolic syndrome, such as obesity and diabetes (Turnbaugh et al., 2006). In addition, studies have found that *Lactobacillus paracasei* X11 can restore the ratio of F/B in the gut microbiota of gout mice and improve the structure and function of the gut microbiota (Cao J. et al., 2022). Therefore, we speculated that the decrease in the ratio of F/B caused by alcohol could contribute to the exacerbation of gout symptoms. At the genus level, the treatment of high concentrations of alcohol significantly increased the relative abundances of *Parasutterella* and *Alistipes* in mice. *Parasutterella*, a core member of the gut microbiota in both human and mouse, is closely associated with the development of inflammatory bowel disease (Blasco-Baque et al., 2017). Metabolomic studies have shown that *Parasutterella* affects the metabolism of aromatic amino acids, bilirubin, purines, and bile acids (Ju et al., 2019). Furhtermore, a significant increase was revealed in the relative abundance of *Parasutterella* in a chronic hepatic injury model induced by alcohol in mice (Sang et al., 2021). Moreover, feeding high-fat food to gout mice was also detected to increase the relative abundance of *Parasutterella*, exacerbating the symptoms of gout (Lin et al., 2020). Bacteroidota are often associated with chronic intestinal inflammation (Parker et al., 2020), while *Alistipes* is a genus of the phylum Bacteroidota. Previous studies have revealed the associations between *Alistipes* and various medical disorders, e.g., hepatic fibrosis, colorectal cancer, and other potential diseases (Moschen et al., 2016; Rau et al., 2018). Furthermore, *Alistipes* uses its unique way of fermentation of amino acids, i.e., putrefaction, which plays a crucial role in inflammation and diseases (Kaur et al., 2017). An increase in the relative abundance of *Alistipes* has also been observed in alcohol-intoxicated mice (Wang et al., 2018). Additionally, it has been found that the increased relative abundance of *Alistipes* could aggravate gout symptoms by regulating the purine metabolism (Shirvani-Rad et al., 2023).

### 4.2. Effects of intake of high concentration of alcohol on metabolism in mice

Our results showed that consumption of high concentrations of alcohol significantly increased the levels of lipopolysaccharide biosynthesis, riboflavin metabolism, phenylalanine metabolism, and arginine and proline metabolism. Lipopolysaccharide has been revealed with many harmful effects and has worsen the disease progression by enhancing the release of inflammatory mediators (Schaffert et al., 2009). Recent studies showed that the bacteria releasing lipopolysaccharide could participate in the pathogenesis of gout by stimulating the immune system (Shirvani-Rad et al., 2023). Furthermore, studies have shown that phenylalanine promotes the alcohol-induced oxidative stress (Hu et al., 2017), while metabolomic studies have revealed a significantly positive correlation between the level of plasma phenylalanine and gout in humans (Mahbub et al., 2017). A recent study found that fructose could aggravate the colonic inflammation by inducing dysregulation of arginine and proline metabolism (Song et al., 2023). Purine metabolism disorder could result in a significant amount of UA production, which in turn affects the progression of gout (Kuo et al., 2015). The riboflavin metabolism in gut microbiota is considered a function involved in purine metabolism (Jiménez et al., 2005), which could lead to higher levels of UA. Furthermore, metabolic studies have shown that alcohol consumption could lead to increased level of serum lactat, thereby blocking the renal excretion of urate salts and potentially leading to increased level of UA (Choi et al., 2004).

Previous studies have demonstrated that *Parasutterella* and *Alistipes* have been implicated in the pathogenesis of constipation and heart disease, respectively, through their impact on lipopolysaccharide biosynthesis and exacerbation of inflammatory responses (Wan et al., 2019; Yang et al., 2021). Studies have shown that taking the new type of synbiotics can lower the relative level of *Parasutterella*, thereby reducing the relative abundance of lipopolysaccharide biosynthesis, as well as significantly reduces the levels of serum LPS, TNF-α, and IL-6, to inhibit the inflammatory response and improve constipation in rats (Yang et al., 2021). In addition, associations between *Parasutterella* and *Alistipes* with phenylalanine metabolism have been detected in ulcerative colitis and brain-related diseases such as Alzheimer’s disease and Parkinson’s disease (Cao C. et al., 2022; Eicher and Mohajeri, 2022). Bacteria with pro-inflammatory characteristics (*Alistipes*) may be involved in microglial cell activation and neuroinflammatory responses by increasing the production of pro-inflammatory cytokines through the modulation of phenylalanine metabolism, thereby contributing to the pathogenesis of brain-related diseases (Eicher and Mohajeri, 2022). Furthermore, in an alcoholic hepatic injury mouse model, an association between *Parasutterella* and riboflavin metabolism was discovered (Lv et al., 2022). Our study revealed no significant effect of high-concentration alcohol consumption on purine metabolism. However, significantly positive correlations were detected among the key bacterial taxa (i.e., *Parasutterella* and *Alistipes*), UA, IL-1β, IL-6, TNF-α, and MPO, as well as the levels of four metabolic pathways (i.e., lipopolysaccharide biosynthesis, riboflavin metabolism, phenylalanine metabolism, and arginine and proline metabolism) in gout mice (Figure 7A). Therefore, it was speculated that high-concentration alcohol consumption alters the taxonomic structure of the gut microbiota and promotes an inflammatory response that could exacerbate gout symptoms. Further studies are needed to provide more evidence to support these speculations.

Considering that both ADA and XOD affect the purine metabolism, we further investigated the enzymatic activation with ADA and XOD in mouse hepatic to explore the exacerbation of gout symptoms by alcohol via purine metabolism. Although our results showed that high alcohol consumption slightly increased the activation of ADA and XOD in mouse hepatic, the differences were not statistically significant due to the small sample size. We hypothesized that this might be related to the lower concentration of alcohol consumed and the shorter treatment time. In the high concentration alcohol group, we found that both 10 and 20% alcohol intake significantly exacerbated acute gouty arthritis symptoms and related clinical indicators, and it was difficult to differentiate the differences between the high concentration alcohol groups. Therefore, we focused on the joint effect of 10 and 20% alcohol intake. Further experimental studies are needed to analyze the differences between 10 and 20% alcohol intake. Notably, while treatment of high-concentration alcohol increased the diversity of the gut microbiota, it significantly reduced the negative correlation between the gut microbiota. We hypothesize that the decrease in negative correlation between bacteria may be responsible for the increased diversity of the gut microbiota. However, our results still need to be validated by further studies. In addition, although our research results indicate that low-concentration alcohol treatment does not have a significant impact on the gut microbiota of gout mice, it also does not have an effect in improving gout symptoms. Therefore, it is highly recommended that the alcohol consumption be avoided during the acute phase of gout.

## 5. Conclusion

In conclusion, our results suggested that high concentrations of alcohol altered the gut microbiota structure in gout mice induced by MSU crystals, possibly exacerbating gout symptoms by enhancing the pro-inflammatory pathways. These results recommended that alcohol consumption should be avoided during the acute phase of gout. Our study provides novel insights into the exacerbation of gouty arthritis by alcohol consumption.

## Data availability statement

The datasets presented in this study can be found in online repositories. The names of the repository/repositories and accession number(s) can be found in the article/Supplementary material.

## Ethics statement

The animal study was approved by the Animal Ethics Review Board of the Provincial Hospital of Shandong First Medical University approved this study (Permit No. 2022-025). The study was conducted in accordance with the local legislation and institutional requirements.

## Author contributions

YF: data curation, methodology, writing – original draft. HS: methodology, software, validation, writing – original draft. RZ: data curation, investigation, writing – original draft. JT: formal analysis, project administration, writing – original draft. RS: data curation, methodology, writing – original draft. YS: conceptualization, formal analysis, funding acquisition, investigation, supervision, validation, writing – review and editing. DW: conceptualization, data curation, funding acquisition, investigation, writing – review and editing.

## Funding

The author(s) declare financial support was received for the research, authorship, and/or publication of this article. This study was financially supported by the National Natural Science Foundation of China (81972057 and 82172313) and the Major Innovation Project of Shandong Province (2021GXGC011305).

## Conflict of interest

The authors declare that the research was conducted in the absence of any commercial or financial relationships that could be construed as a potential conflict of interest.

## Publisher’s note

All claims expressed in this article are solely those of the authors and do not necessarily represent those of their affiliated organizations, or those of the publisher, the editors and the reviewers. Any product that may be evaluated in this article, or claim that may be made by its manufacturer, is not guaranteed or endorsed by the publisher.

## Footnotes

^1^http://www.arb-silva.de/

^2^http://sourceforge.net/projects/rdpclassifier/

^3^https://www.genome.jp/kegg/

^4^https://www.ncbi.nlm.nih.gov/sra/

## References

[ref1] Ankli B. Daikeler T. (2023). Update Gicht [update gout]. Ther. Umsch. 80, 17–26. doi: 10.1024/0040-5930/a001402, PMID: 36659847

[ref2] Barbería-Latasa M. Gea A. Martínez-González M. A. (2022). Alcohol, drinking pattern, and chronic disease. Nutrients 14:1954. doi: 10.3390/nu14091954, PMID: 35565924 PMC9100270

[ref3] Blasco-Baque V. Coupé B. Fabre A. Handgraaf S. Gourdy P. Arnal J. F.. (2017). Associations between hepatic miRNA expression, liver triacylglycerols and gut microbiota during metabolic adaptation to high-fat diet in mice. Diabetologia 60, 690–700. doi: 10.1007/s00125-017-4209-3, PMID: 28105518 PMC6518927

[ref4] Cao J. Liu Q. Hao H. Bu Y. Tian X. Wang T.. (2022). *Lactobacillus paracasei* X11 ameliorates hyperuricemia and modulates gut microbiota in mice. Front. Immunol. 13:940228. doi: 10.3389/fimmu.2022.940228, PMID: 35874662 PMC9296831

[ref5] Cao C. Wang L. Ai C. Gong G. Wang Z. Huang L.. (2022). Impact of *Lycium barbarum* arabinogalactan on the fecal metabolome in a DSS-induced chronic colitis mouse model. Food Funct. 13, 8703–8716. doi: 10.1039/d2fo01283a, PMID: 35912853

[ref6] Cheng M. Tan B. Wu X. Liao F. Wang F. Huang Z. (2021). Gut microbiota is involved in alcohol-induced osteoporosis in young and old rats through immune regulation. Front. Cell. Infect. Microbiol. 11:636231. doi: 10.3389/fcimb.2021.636231, PMID: 34336709 PMC8317599

[ref7] Choi H. K. Atkinson K. Karlson E. W. Willett W. Curhan G. (2004). Alcohol intake and risk of incident gout in men: a prospective study. Lancet 363, 1277–1281. doi: 10.1016/S0140-6736(04)16000-5, PMID: 15094272

[ref8] Compare D. Rocco A. Sanduzzi Zamparelli M. Nardone G. (2016). The gut Bacteria-driven obesity development. Dig. Dis. 34, 221–229. doi: 10.1159/000443356, PMID: 27028448

[ref9] Dubinkina V. B. Tyakht A. V. Odintsova V. Y. Yarygin K. S. Kovarsky B. A. Pavlenko A. V.. (2017). Links of gut microbiota composition with alcohol dependence syndrome and alcoholic liver disease. Microbiome 5:141. doi: 10.1186/s40168-017-0359-2, PMID: 29041989 PMC5645934

[ref10] Eicher T. P. Mohajeri M. H. (2022). Overlapping mechanisms of action of brain-active Bacteria and bacterial metabolites in the pathogenesis of common brain diseases. Nutrients 14:2661. doi: 10.3390/nu14132661, PMID: 35807841 PMC9267981

[ref11] Feng Y. Yu Y. Chen Z. Wang L. Ma J. Bai X.. (2022). Effects of β-Carotin and green tea powder diets on alleviating the symptoms of gouty arthritis and improving gut microbiota in C57BL/6 mice. Front. Microbiol. 13:837182. doi: 10.3389/fmicb.2022.837182, PMID: 35145506 PMC8821968

[ref12] Galozzi P. Bindoli S. Doria A. Oliviero F. Sfriso P. (2021). Autoinflammatory features in gouty arthritis. J. Clin. Med. 10:1880. doi: 10.3390/jcm10091880, PMID: 33926105 PMC8123608

[ref13] Guo Z. Zhang J. Wang Z. Ang K. Y. Huang S. Hou Q.. (2016). Intestinal microbiota distinguish gout patients from healthy humans. Sci. Rep. 6:20602. doi: 10.1038/srep20602, PMID: 26852926 PMC4757479

[ref14] Hendriks H. F. J. (2020). Alcohol and human health: what is the evidence? Annu. Rev. Food Sci. Technol. 11, 1–21. doi: 10.1146/annurev-food-032519-051827 32209032

[ref15] Hu Q. Wei J. Liu Y. Fei X. Hao Y. Pei D.. (2017). Discovery and identification of potential biomarkers for alcohol-induced oxidative stress based on cellular metabolomics. Biomed. Chromatogr. 31:e3907. doi: 10.1002/bmc.3907, PMID: 27925248

[ref16] Jiménez A. Santos M. A. Pompejus M. Revuelta J. L. (2005). Metabolic engineering of the purine pathway for riboflavin production in ashbya gossypii. Appl. Environ. Microbiol. 71, 5743–5751. doi: 10.1128/AEM.71.10.5743-5751.2005, PMID: 16204483 PMC1265981

[ref17] Ju T. Kong J. Y. Stothard P. Willing B. P. (2019). Defining the role of Parasutterella, a previously uncharacterized member of the core gut microbiota. ISME J. 13, 1520–1534. doi: 10.1038/s41396-019-0364-5, PMID: 30742017 PMC6776049

[ref18] Kaur H. Das C. Mande S. S. (2017). In silico analysis of putrefaction pathways in Bacteria and its implication in colorectal Cancer. Front. Microbiol. 8:2166. doi: 10.3389/fmicb.2017.02166, PMID: 29163445 PMC5682003

[ref19] Kogut M. H. Lee A. Santin E. (2020). Microbiome and pathogen interaction with the immune system. Poult. Sci. 99, 1906–1913. doi: 10.1016/j.psj.2019.12.011, PMID: 32241470 PMC7587753

[ref20] Kuo C. F. Grainge M. J. Zhang W. Doherty M. (2015). Global epidemiology of gout: prevalence, incidence and risk factors. Nat. Rev. Rheumatol. 11, 649–662. doi: 10.1038/nrrheum.2015.91 26150127

[ref21] Larsson S. C. Wallin A. Wolk A. Markus H. S. (2016). Differing association of alcohol consumption with different stroke types: a systematic review and meta-analysis. BMC Med. 14:178. doi: 10.1186/s12916-016-0721-4, PMID: 27881167 PMC5121939

[ref22] Larsson S. C. Wallin A. Wolk A. Markus H. S. Islami F. Fedirko V.. (2013). Light alcohol drinking and cancer: a meta-analysis. Ann. Oncol. 14, 301–308. doi: 10.1186/s12916-016-0721-4, PMID: 22910838

[ref23] Leclercq S. Matamoros S. Cani P. D. Neyrinck A. M. Jamar F. Stärkel P.. (2014). Intestinal permeability, gut-bacterial dysbiosis, and behavioral markers of alcohol-dependence severity. Proc. Natl. Acad. Sci. U. S. A. 111, E4485–E4493. doi: 10.1073/pnas.1415174111, PMID: 25288760 PMC4210345

[ref24] Li X. H. Yu F. F. Zhou Y. H. He J. (2016). Association between alcohol consumption and the risk of incident type 2 diabetes: a systematic review and dose-response meta-analysis. Am. J. Clin. Nutr. 103, 818–829. doi: 10.3945/ajcn.115.114389, PMID: 26843157

[ref25] Lin X. Shao T. Wen X. Wang M. Wen C. He Z. (2020). Combined effects of MSU crystals injection and high fat-diet feeding on the establishment of a gout model in C57BL/6 mice. Adv. Rheumatol. 60:52. doi: 10.1186/s42358-020-00155-3, PMID: 33148336

[ref26] Liu Y. R. Tantoh D. M. Lin C. C. Hsiao C. H. Liaw Y. P. (2021). Risk of gout among Taiwanese adults with ALDH-2 rs671 polymorphism according to BMI and alcohol intake. Arthritis Res. Ther. 23:115. doi: 10.1186/s13075-021-02497-9, PMID: 33858492 PMC8048165

[ref27] Lou X. J. Wang Y. Z. Lei S. S. He X. Lu T. T. Zhan L. H.. (2020). Beneficial effects of macroporous resin extract of Dendrobium candidum leaves in rats with hyperuricemia induced by a high-purine diet. Evid. Based Complement. Alternat. Med. 2020:3086106. doi: 10.1155/2020/3086106, PMID: 32089717 PMC7023721

[ref28] Lv X. C. Wu Q. Cao Y. J. Lin Y. C. Guo W. L. Rao P. F.. (2022). Ganoderic acid a from Ganoderma lucidum protects against alcoholic liver injury through ameliorating the lipid metabolism and modulating the intestinal microbial composition. Food Funct. 13, 5820–5837. doi: 10.1039/d1fo03219d, PMID: 35543349

[ref29] Mahbub M. H. Yamaguchi N. Takahashi H. Hase R. Amano H. Kobayashi-Miura M.. (2017). Alteration in plasma free amino acid levels and its association with gout. Environ. Health Prev. Med. 22:7. doi: 10.1186/s12199-017-0609-8, PMID: 29165113 PMC5664792

[ref30] Moschen A. R. Gerner R. R. Wang J. Klepsch V. Adolph T. E. Reider S. J.. (2016). Lipocalin 2 protects from inflammation and tumorigenesis associated with gut microbiota alterations. Cell Host Microbe 19, 455–469. doi: 10.1016/j.chom.2016.03.007, PMID: 27078067

[ref31] Natividad J. M. Pinto-Sanchez M. I. Galipeau H. J. Jury J. Jordana M. Reinisch W.. (2015). Ecobiotherapy rich in Firmicutes decreases susceptibility to colitis in a humanized Gnotobiotic mouse model. Inflamm. Bowel Dis. 21, 1883–1893. doi: 10.1097/MIB.0000000000000422, PMID: 26060932

[ref32] Nemoto N. Takeda Y. Nara H. Araki A. Gazi M. Y. Takakubo Y.. (2020). Analysis of intestinal immunity and flora in a collagen-induced mouse arthritis model: differences during arthritis progression. Int. Immunol. 32, 49–56. doi: 10.1093/intimm/dxz058, PMID: 31562738

[ref33] Neogi T. Chen C. Niu J. Chaisson C. Hunter D. J. Zhang Y. (2014). Alcohol quantity and type on risk of recurrent gout attacks: an internet-based case-crossover study. Am. J. Med. 127, 311–318. doi: 10.1016/j.amjmed.2013.12.019, PMID: 24440541 PMC3991555

[ref34] Parker B. J. Wearsch P. A. Veloo A. C. M. Rodriguez-Palacios A. (2020). The genus Alistipes: gut Bacteria with emerging implications to inflammation, Cancer, and mental health. Front. Immunol. 11:906. doi: 10.3389/fimmu.2020.00906, PMID: 32582143 PMC7296073

[ref35] Postler T. S. Ghosh S. (2017). Understanding the holobiont: how microbial metabolites affect human health and shape the immune system. Cell Metab. 26, 110–130. doi: 10.1016/j.cmet.2017.05.008, PMID: 28625867 PMC5535818

[ref36] Qin Y. Havulinna A. S. Liu Y. Jousilahti P. Ritchie S. C. Tokolyi A.. (2022). Combined effects of host genetics and diet on human gut microbiota and incident disease in a single population cohort. Nat. Genet. 54, 134–142. doi: 10.1038/s41588-021-00991-z, PMID: 35115689 PMC9883041

[ref37] Rau M. Rehman A. Dittrich M. Groen A. K. Hermanns H. M. Seyfried F.. (2018). Fecal SCFAs and SCFA-producing bacteria in gut microbiome of human NAFLD as a putative link to systemic T-cell activation and advanced disease. United European Gastroenterol. J. 6, 1496–1507. doi: 10.1177/2050640618804444, PMID: 30574320 PMC6297934

[ref38] Ruiz-Miyazawa K. W. Staurengo-Ferrari L. Mizokami S. S. Domiciano T. P. Vicentini F. T. M. C. Camilios-Neto D.. (2017). Quercetin inhibits gout arthritis in mice: induction of an opioid-dependent regulation of inflammasome. Inflammopharmacology 25, 555–570. doi: 10.1007/s10787-017-0356-x, PMID: 28508104

[ref39] Sang L. Kang K. Sun Y. Li Y. Chang B. (2021). FOXO4 ameliorates alcohol-induced chronic liver injury via inhibiting NF-κB and modulating gut microbiota in C57BL/6J mice. Int. Immunopharmacol. 96:107572. doi: 10.1016/j.intimp.2021.107572, PMID: 33798806

[ref40] Schaffert C. S. Duryee M. J. Hunter C. D. Hamilton B. C. DeVeney A. L. Huerter M. M.. (2009). Alcohol metabolites and lipopolysaccharide: roles in the development and/or progression of alcoholic liver disease. World J. Gastroenterol. 15, 1209–1218. doi: 10.3748/wjg.15.1209, PMID: 19291821 PMC2658861

[ref41] Shirvani-Rad S. Khatibzade-Nasari N. Ejtahed H. S. Larijani B. (2023). Exploring the role of gut microbiota dysbiosis in gout pathogenesis: a systematic review. Front. Med. 10:1163778. doi: 10.3389/fmed.2023.1163778, PMID: 37265486 PMC10230090

[ref42] Singh J. A. Gaffo A. (2020). Gout epidemiology and comorbidities. Semin. Arthritis Rheum. 50, S11–S16. doi: 10.1016/j.semarthrit.2020.04.008 32620196

[ref43] So A. K. Martinon F. (2017). Inflammation in gout: mechanisms and therapeutic targets. Nat. Rev. Rheumatol. 13, 639–647. doi: 10.1038/nrrheum.2017.155 28959043

[ref44] Song G. Gan Q. Qi W. Wang Y. Xu M. Li Y. (2023). Fructose stimulated colonic arginine and proline metabolism dysbiosis, altered microbiota and aggravated intestinal barrier dysfunction in DSS-induced colitis rats. Nutrients 15:782. doi: 10.3390/nu15030782, PMID: 36771488 PMC9921751

[ref45] Terkeltaub R. (2017). What makes gouty inflammation so variable? BMC Med. 15, 1–10. doi: 10.1186/s12916-017-0922-5 28818081 PMC5561591

[ref46] Tu H. P. Tung Y. C. Tsai W. C. Lin G. T. Ko Y. C. Lee S. S. (2017). Alcohol-related diseases and alcohol dependence syndrome is associated with increased gout risk: a nationwide population-based cohort study. Jt. Bone Spine 84, 189–196. doi: 10.1016/j.jbspin.2016.02.024, PMID: 27238189

[ref47] Turnbaugh P. J. Ley R. E. Mahowald M. A. Magrini V. Mardis E. R. Gordon J. I. (2006). An obesity-associated gut microbiome with increased capacity for energy harvest. Nature 444, 1027–1031. doi: 10.1038/nature05414, PMID: 17183312

[ref48] Vieira A. T. Macia L. Galvão I. Martins F. S. Canesso M. C. Amaral F. A.. (2015). A role for gut microbiota and the metabolite-sensing receptor GPR43 in a murine model of gout. Arthritis Rheumatol. 67, 1646–1656. doi: 10.1002/art.39107 25914377

[ref49] Wan Y. Wang F. Yuan J. Li J. Jiang D. Zhang J.. (2019). Effects of dietary fat on gut microbiota and faecal metabolites, and their relationship with cardiometabolic risk factors: a 6-month randomised controlled-feeding trial. Gut 68, 1417–1429. doi: 10.1136/gutjnl-2018-317609, PMID: 30782617

[ref50] Wang L. Fouts D. E. Stärkel P. Hartmann P. Chen P. Llorente C.. (2016). Intestinal REG3 lectins protect against alcoholic steatohepatitis by reducing mucosa-associated microbiota and preventing bacterial translocation. Cell Host Microbe 19, 227–239. doi: 10.1016/j.chom.2016.01.003, PMID: 26867181 PMC4786170

[ref51] Wang G. Liu Q. Guo L. Zeng H. Ding C. Zhang W.. (2018). Gut microbiota and relevant metabolites analysis in alcohol dependent mice. Front. Microbiol. 9:1874. doi: 10.3389/fmicb.2018.01874, PMID: 30158912 PMC6104187

[ref52] Yang Z. Ye S. Xu Z. Su H. Tian X. Han B.. (2021). Dietary synbiotic ameliorates constipation through the modulation of gut microbiota and its metabolic function. Food Res. Int. 147:110569. doi: 10.1016/j.foodres.2021.110569, PMID: 34399543

[ref53] Zhang J. Wang M. Qi X. Shi L. Zhang J. Zhang X.. (2021). Predicting the postmortem interval of burial cadavers based on microbial community succession. Forensic. Sci. Int. Genet. 52:102488. doi: 10.1016/j.fsigen.2021.102488, PMID: 33667880

