# Supplementary material for: Effects of alcohol on the symptoms of gouty arthritis and taxonomic structure of gut microbiota in C57BL/6 mice
Source: Front Microbiol. 2023 Sep 13;14:1257701. doi: 10.3389/fmicb.2023.1257701 (PMC10525330; doi:10.3389/fmicb.2023.1257701)
Supplement: Supplementary file 1 [file Image_1.pdf]

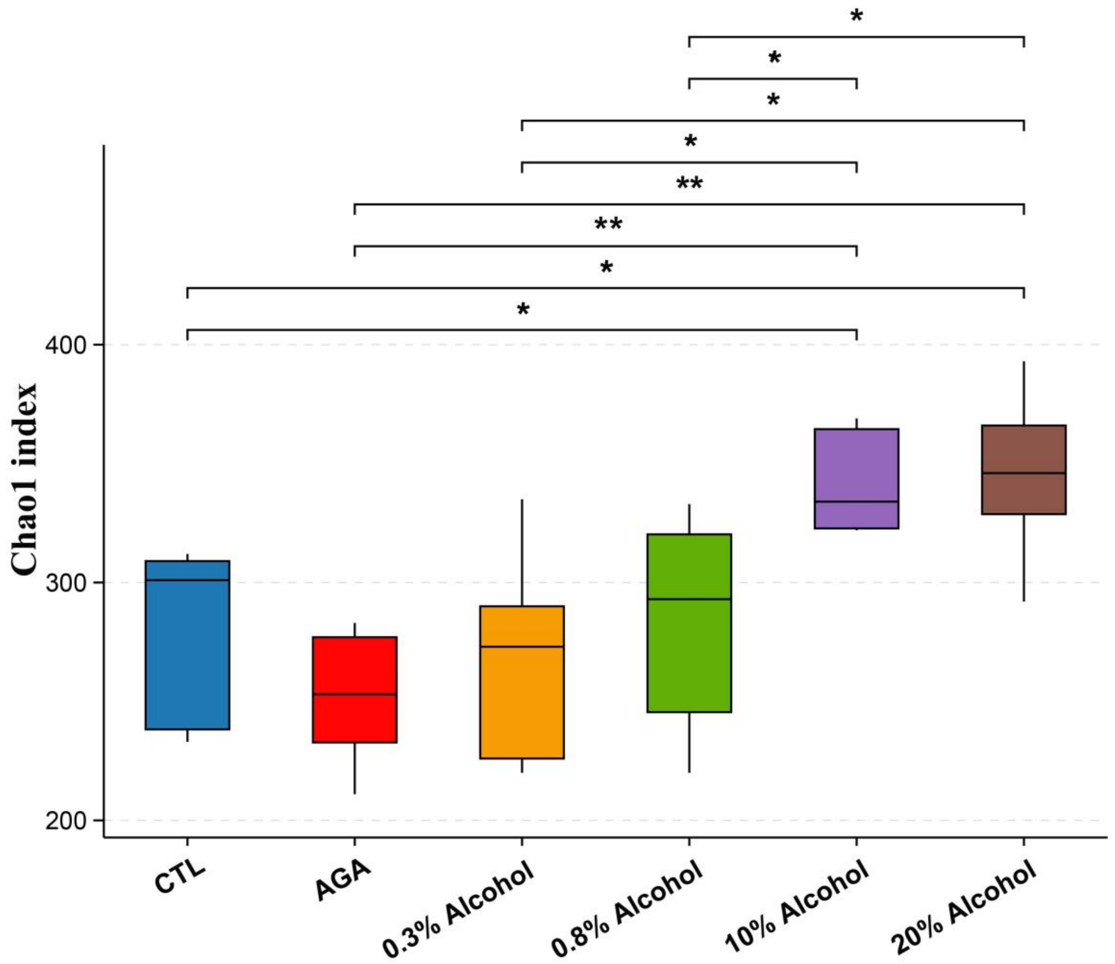

Supplementary Figure 1. Alpha diversity analysis of the intestinal microbiota in six groups of mice. Data are presented as mean ± standard deviation. Statistical differences are determined by $P < 0.05$ (*) and $P < 0.01$ (**), respectively.

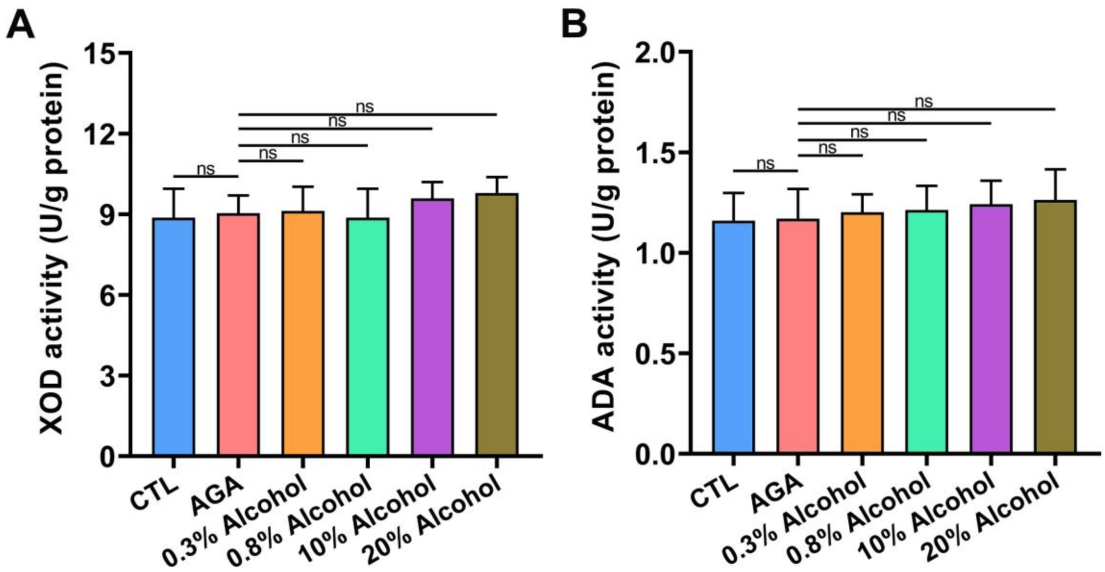

Supplementary Figure 2. Effect of alcohol on the activities of enzymes related to purine metabolism in mouse hepatic tissue. (A) Comparison of changes in the enzymatic levels of xanthine oxidase (XOD) in the hepatic tissue of six groups of mice based on ELISA. (B) Comparison of changes in the enzymatic levels of adenosine deaminase (ADA) in the hepatic tissue of six groups of mice based on ELISA. Data are presented as mean ± standard deviation. “ns” indicates no statistical significance.
